# Role of Naltrexone Plus Bupropion in Eating Behavior Adjustment: A Systematic Review and Meta-Analysis of Randomized Controlled Trials

**DOI:** 10.1192/j.eurpsy.2025.493

**Published:** 2025-08-26

**Authors:** D. F. Holanda, M. G. Ruelas, F. R. de Lima, G. H. S. da Silva, C. M. C. G. Q. Campos, B. S. Pinto

**Affiliations:** 1 Medicine, Federal University of Amazonas, Manaus, Brazil; 2 Instituto de Investigación Nutricional, Lima, Peru; 3 Independent practice, São Paulo; 4 Medicine, Catholic University of Pernambuco, Recife, Brazil

## Abstract

**Introduction:**

Binge-eating disorder (BED) is a significant global health challenge associated with obesity and psychological issues. The combination of Naltrexone-Bupropion (NB) has emerged as a promising pharmacological approach for managing eating behaviors.

**Objectives:**

This meta-analysis aims to evaluate the efficacy and safety of Naltrexone-Bupropion compared to placebo in managing eating behaviors, focusing on weight loss, binge-eating frequency, eating disorder psychopathology, quality of life, and adverse effects.

**Methods:**

PubMed, Embase and Cochrane databases were searched for randomized controlled trials (RCT) comparing NB versus placebo for BED. Primary endpoints were weight loss and binge-eating frequency. Secondary endpoints included eating disorder psychopathology, depression, quality of life, food cravings, and adverse effects.The mean differences (MD) were applied with their 95% confidence intervals (95%CIs) for continuous outcomes, using a random-effects model. We used RevMan 5.4.1 for statistical analyses. Heterogeneity was assessed using the I² statistic.

**Results:**

Five RCTs with 2,466 adult participants (mean age 46.5 years, BMI 21.5-50 kg/m²) were included. NB was associated with a statistically significant reduction in weight loss percentage compared to placebo (MD -3.67%, 95% CI [-4.30; -3.03], I²=98%; Figure 1). However, no significant differences were found between NB and placebo in reducing binge-eating episodes(SMD 0.02, 95% CI [-0.30; 0.34], I2 =0%, Figure 2) , improving eating disorder psychopathology, alleviating depression, or decreasing food cravings. Although NB showed some benefits in improving the quality of life, the results were not statistically significant. NB was associated with a higher risk of adverse effects, including nausea, headache, constipation, dizziness, vomiting, insomnia, and dry mouth. The certainty of the evidences is in the Summary of findings (SOF) of GRADE evaluation (Figure 3). After leave-one-out sensitivity analysis, no single study was found to influence the effect estimate or drive heterogeneity excessively.

**Image 1:**

**Figure:**
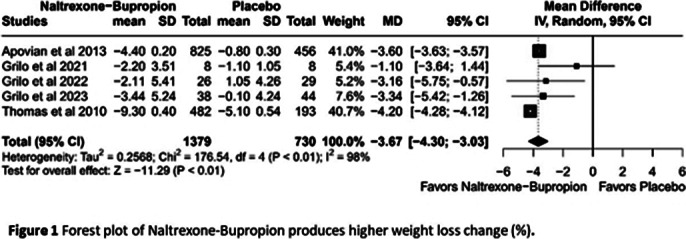

**Image 2:**

**Figure:**
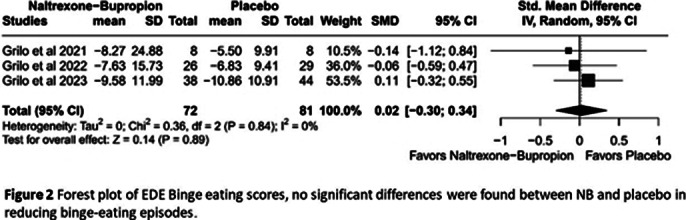

**Image 3:**

**Figure:**
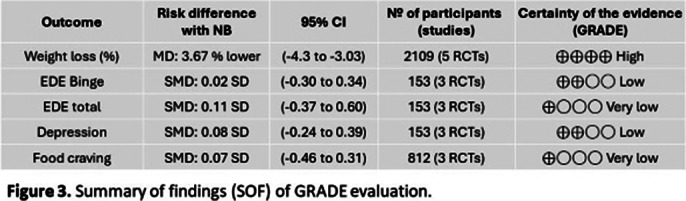

**Conclusions:**

NB demonstrated efficacy for weight loss in individuals with eating behavior issues but showed no significant benefits for core eating disorder symptoms. The higher risk of adverse effects necessitates careful consideration in clinical decision-making. Further research is needed to determine optimal patient populations, treatment duration, and strategies to mitigate adverse effects.

**Disclosure of Interest:**

None Declared

